# The SIX Family of Transcription Factors: Common Themes Integrating Developmental and Cancer Biology

**DOI:** 10.3389/fcell.2021.707854

**Published:** 2021-08-19

**Authors:** Logan Meurer, Leonard Ferdman, Beau Belcher, Troy Camarata

**Affiliations:** ^1^Department of Basic Sciences, NYIT College of Osteopathic Medicine at Arkansas State University, Jonesboro, AR, United States; ^2^Department of Biological Sciences, Arkansas State University, Jonesboro, AR, United States

**Keywords:** transcription factor, SIX genes, developmental biology, cancer, congenital disease

## Abstract

The *sine oculis* (SIX) family of transcription factors are key regulators of developmental processes during embryogenesis. Members of this family control gene expression to promote self-renewal of progenitor cell populations and govern mechanisms of cell differentiation. When the function of *SIX* genes becomes disrupted, distinct congenital defects develops both in animal models and humans. In addition to the embryonic setting, members of the SIX family have been found to be critical regulators of tumorigenesis, promoting cell proliferation, epithelial-to-mesenchymal transition, and metastasis. Research in both the fields of developmental biology and cancer research have provided an extensive understanding of SIX family transcription factor functions. Here we review recent progress in elucidating the role of *SIX* family genes in congenital disease as well as in the promotion of cancer. Common themes arise when comparing SIX transcription factor function during embryonic and cancer development. We highlight the complementary nature of these two fields and how knowledge in one area can open new aspects of experimentation in the other.

## Introduction

**FIGURE 1:**
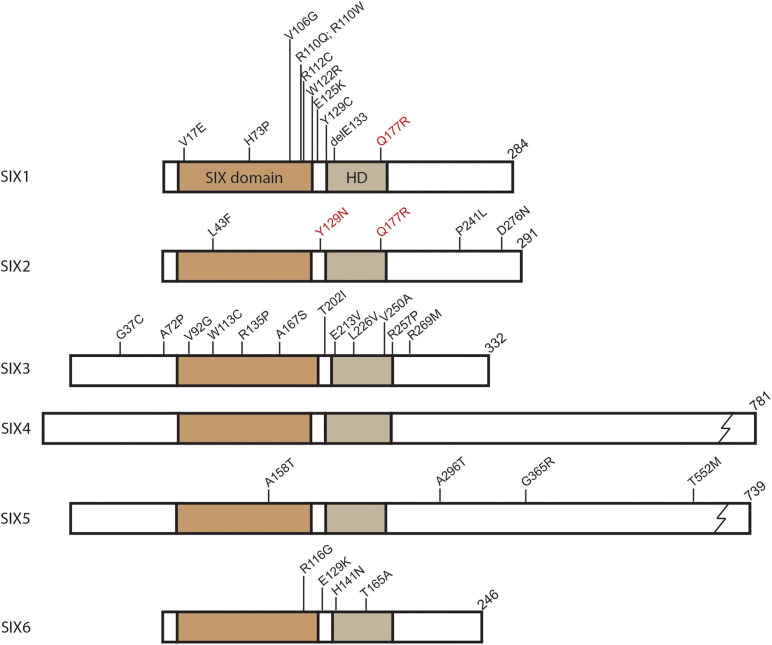
SIX protein domain schematic and identified human mutations. Position of conserved domains shown for SIX domain and Homeodomain (HD). Mutations identified in *SIX* genes related to congenital conditions are labeled at their relative amino acid position. In addition to the mutations shown, single allele deletions have been identified for *SIX2*, *SIX3*, and *SIX6*. *SIX1* and *SIX2* hyperactive mutations associated with Wilms tumor denoted in red. Common identified *SIX3* mutations shown. For a complete list of *SIX3* mutations identified in holoprosencephaly see Lacbawan et al. (2009).

The *sine oculis* (SIX) homeobox family of transcription factors play important developmental roles in a wide range of species from fruit flies to humans. The founding member, *sine oculis* (*so*), was first identified in *Drosophila melanogaster* where it was discovered to be required for compound eye formation (Cheyette et al., 1994; Serikaku and O’Tousa, 1994). Subsequent research in fruit flies identified two additional SIX genes, *optix*, and *DSix4* (reviewed in Kawakami et al., 2000). All three transcription factors were found to share a conserved N-terminal SIX domain adjacent to a homeodomain (HD), which function as protein-protein and DNA binding domains, respectively (Figure 1; Kawakami et al., 2000). Gene duplication during evolution expanded the *SIX* family of genes and created three subfamilies in vertebrates, which are composed of the *so* subfamily (*Six1* and *Six2*), the *optix* subfamily (*Six3* and *Six6*), and the *DSix4* subfamily (*Six4* and *Six5*). The vertebrate orthologs contain the same SIX and HD domains as the ancestral *Drosophila* proteins with significant amino acid sequence identity between the functional domains of family members. For example, the mouse SIX domain amino acid identity ranges from 63 to 93% while sequence identity in the HD ranges from 59 to 98% between family members, with Six3, Six4, and Six5 showing the most divergence. For a more complete review of SIX family protein structure and sequence comparisons see Kawakami et al. (2000) and Kumar (2009).

In vertebrates, *SIX* genes play critical roles in tissue formation and organogenesis, such as for the head, ear, retina, nose, brain, skeletal muscle, and kidney (Oliver et al., 1995; Ohto et al., 1998; Jean et al., 1999; Kobayashi et al., 2001; Laclef et al., 2003; Lagutin et al., 2003; Li et al., 2003; Xu et al., 2003; Self et al., 2006). In these tissues, the SIX family of transcription factors function as regulators of progenitor cell maintenance and differentiation. They can act as transcriptional activators or repressors depending on interactions with other highly conserved regulators including Paired-box (Pax), Eyes absent (Eya), Dachshund (Dach), and Groucho (Grg) proteins (see reviews Kawakami et al., 2000; Kumar, 2009). Correlating with their important functions during embryogenesis, several congenital defects in humans are associated with mutations in *SIX* genes. In cancer, *SIX* genes have been found to be ectopically- or over-expressed and experimental interrogation suggests SIX proteins can drive disease pathogenesis. This review will focus on recent developments on *SIX* gene function and regulation in order to provide insight into congenital diseases along with how *SIX* genes become exploited in the context of cancer formation. Common themes emerge when comparing roles of *SIX* genes in developmental and cancer biology which relate to progenitor cell maintenance, cell behavior, and regulation; generating new questions and opportunities for research in each field.

## Congenital Disease and Associated Developmental Biology

Members of the SIX family of transcription factors are widely expressed in the developing vertebrate embryo where they play important regulatory roles in tissue and organ formation. Unsurprisingly, several human congenital conditions have been associated with haploinsufficiency or hypomorphic mutations in *SIX* genes (Figure 1 and Table 1). Many human *SIX* gene mutations were located within the SIX domain or homeodomain, which were suggestive of impaired protein-protein interactions or DNA binding. However, mutations have been detected outside of these domains and their functional significance remains unclear (Figure 1). Mutations in human *SIX1* and *SIX2* have been associated with multiple congenital disorders such as branchio-oto-renal syndrome (BOR), renal dysplasia, hearing loss, and frontonasal dysplasia syndrome (Ruf et al., 2004; Kochhar et al., 2008; Weber et al., 2008; Mosrati et al., 2011; Guan et al., 2016; Hufnagel et al., 2016). BOR is an autosomal dominant disorder that is characterized by a triad of clinical symptoms including branchial arch defects, hearing loss, and renal abnormalities (Melnick et al., 1976). The most commonly identified mutations in BOR are in the SIX1 binding partner *EYA1* (Abdelhak et al., 1997), however, mutations have been identified in *SIX1* that disrupt DNA binding or impact the ability to form a functional transcriptional complex with EYA1 (Ruf et al., 2004; Patrick et al., 2009). Independent of BOR, dominantly inherited hearing loss has been associated with mutations in both *SIX1* and *SIX2* (Mosrati et al., 2011; Guan et al., 2016). Additionally, *SIX2* mutations were detected in patients with renal hypodysplasia (Weber et al., 2008) and more recently in frontonasal dysplasia syndrome (Hufnagel et al., 2016).

**TABLE 1:** Associated congenital conditions and related mouse model data for SIX family members.

Gene	Associated human congenital disease	Genetic result of human mutation	Mouse embryonic expression	Single gene mouse knock-out phenotype(s)
SIX1	Branchio-oto-renal Syndrome (BOR)	Hypomorph	Lung, otic vesicles, nephric cords/kidney, urinary tract, pharyngeal pouch, olfactory epithelium, mammary gland, gonads; somites/skeletal muscle; secondary heart field	Malformed inner and middle ear, nasal cavity defects, missing thymus, renal hypoplasia or agenesis, reduced skeletal muscle mass
		Disruption of DNA binding		
	Autosomal dominant deafness	Disruption of Eya interactions		
	Wilms tumor	Hyperactivation in Wilms tumor		
SIX2	Renal hypodysplasia	Haploinsufficiency or hypomorph	Kidney, palate, cranial base chondrocytes; secondary heart field	Renal hypoplasia, craniofacial defects
	Frontonasal dysplasia syndrome			
	Autosomal dominant deafness	Hyperactivation in Wilms tumor		
	Wilms tumor			
SIX3	Holoprosencephaly	Haploinsufficiency or hypomorph	Forebrain neurons, retina	Missing head structures anterior to midbrain, eyes, nose
	Schizencephaly			
SIX4	Omphalocele	ND	Kidney, olfactory epithelium, gonads, skeletal muscle	None detected
SIX5	Myotonic dystrophy	Reduced gene expression	Spermatogonia, abdominal wall, skeletal muscle	Cataracts, male reproductive defects
	Branchio-oto-renal Syndrome (BOR)	Potentially reduced DNA binding		
	Omphalocele			
SIX6	Primary open angle glaucoma	Haploinsufficiency or hypomorph	Hypothalamus, retina, pituitary	Hypoplasia of pituitary gland and retina

*See text for details and associated references. ND, not determined.*

The involvement of *SIX1* and *SIX2* in human syndromes is supported by functional studies in animal models. Aspects of BOR are evident in mouse knock-out models as *Six1*^–/–^ null mice fail to develop a thymus and kidney, in addition to having significant defects in structures of the inner ear and nose (Zheng et al., 2003; Xu et al., 2003; Ruf et al., 2004; Ozaki et al., 2004; Zou et al., 2006). Further analysis has shown *Six1* to be required for otic vesicle and cochlea development as well as differentiation of hair cells in the ear (Ozaki et al., 2004; Zhang et al., 2017). Identified human BOR *SIX1* mutations expressed in *Xenopus* embryos disrupted otic vesicle formation and ear morphology, further connecting *SIX1* function to BOR phenotypes (Shah et al., 2020). *Six2* manipulation in animals models has also been informative in its role during development. For example, *Six2* knock-out mice develop renal hypoplasia, where the metaneprhic kidney forms but is significantly smaller than normal (Self et al., 2006; Kobayashi et al., 2008). Mouse *Six2* mutants also display features associated with frontonasal dysplasia. Two independent *Six2* animal models, a genetic knockout and a mutant from an X-ray irradiation screen (*Brachyrrhine*), exhibited defects of the cranial base and cleft palate (Self et al., 2006; Fogelgren et al., 2008; He et al., 2010; Sweat et al., 2020). These phenotypes were similar to a dominantly inherited form of frontonasal dysplasia syndrome linked to a chromosomal deletion of *SIX2* at 2p21 (Hufnagel et al., 2016). *Six1* has also been linked to craniofacial development as disruption of *Six1* function leads to micrognathia and defects of the nose, mandible, and midface structures (Ozaki et al., 2004; Guo et al., 2011; Tavares et al., 2017). Furthermore, both *so* homologs have been shown to participate together in craniofacial development as *Six1*/*Six2* double knockout animals have significantly more severe phenotypes than single mutants, including agenesis of the frontal and parietal bones of the skull (Liu et al., 2019a).

SIX3 and SIX6 of the *optix* subfamily have been associated with specific congenital defects of the developing forebrain in humans. Mutations in *SIX3* result in holoprosencephaly (HPE), a heterogeneous collection of forebrain malformations, with incomplete penetrance due to *SIX3* haploinsufficiency or hypomorphic function (Table 1; Wallis et al., 1999; Lacbawan et al., 2009; Hehr et al., 2010; Stokes et al., 2018). Mutations in additional genes are also known to cause HPE including *Sonic Hedgehog* (*SHH)* and other members of this signaling pathway (Dubourg et al., 2007). Mutations in *SIX3* and *SHH* have both been implicated in schizencephaly, a syndrome which clinically overlaps with HPE (Hehr et al., 2010). *SIX6* has been connected with various eye malformations including anophthalmia and microphthalmia (Gallardo et al., 1999, 2004), primary open angle glaucoma (POAG) (Iglesias et al., 2014; Carnes et al., 2014; Mohanty et al., 2018), and optic disk anomalies and macular atrophy (Yariz et al., 2015). Similar to other family members, mutations such as point mutations and allele deletions in *SIX6* were thought to result in haploinsufficiency or hypomorphic function (Figure 1 and Table 1).

In the mammalian embryo, *Six3* and *Six6* are expressed in derivatives of the anterior neural plate including the hypothalamus, pituitary gland, olfactory placodes, and regions of the developing eye (Oliver et al., 1995; Jean et al., 1999). Functional studies have shown a requirement for *Six3* in forebrain development, for example, Six3 knock-out mice do not form telencephalic or optic vesicles (Lagutin et al., 2003). *Shh*, which has been shown to be involved in forebrain formation and HPE presentations (Shimamura and Rubenstein, 1997; Fuccillo et al., 2004), is directly regulated by Six3 (Jeong et al., 2008; Geng et al., 2008). The Six3-Shh interaction in the anterior neural plate is required to repress Wnt signaling, allowing for proper dorsoventral patterning of the telencephalon (Geng et al., 2008; Jeong et al., 2008; Liu et al., 2010; Carlin et al., 2012). Manipulation of *Six3* expression in animal models has also provided insight into the incomplete penetrance observed in familial HPE. Knock-in of human mutations or the creation of variable expressing hypomorphs in mice has modeled both semilobar and alobar HPE with variable penetrance (Geng et al., 2008, 2016). The ultimate result of haploinsufficiency or hypomorphic alleles of *SIX3* is reduced SHH signaling and defective forebrain formation. Compared to *Six3*, *Six6* was found to be more restricted during forebrain development with expression in the forming hypothalamus, pituitary, and retina (Jean et al., 1999). Disruption of *Six6* function in animal models has led to defects in the visual system ranging from small eye phenotypes in zebrafish to hypoplasia of the neural retina in mice (Li et al., 2002; Iglesias et al., 2014; Carnes et al., 2014; Teotia et al., 2017; Diacou et al., 2018). Conditional knockout of *Six3* or *Six6* have provided additional insights into the roles of the transcription factors in eye development. Neural retina maintenance and differentiation is dependent upon the function of both *Six3* and *Six6* (Zhu et al., 2002; Liu et al., 2006, 2010; Manavathi et al., 2007; Samuel et al., 2016; Takata et al., 2017; Liu and Cvekl, 2017) and both genes are required to repress Wnt signaling during eye development (Diacou et al., 2018). Coordinated and overlapping roles for the two transcription factors has also been implicated in the hypothalamus and pituitary gland where they may regulate the neurons that express gonadotropin-releasing hormone and differentiation of the receptive pituitary gonadotropes (Larder et al., 2011; Xie et al., 2015).

Of the *DSix4* subfamily members, *Six4* and *Six5*, only *SIX5* has thus far been linked to congenital disease in humans (Table 1). Along with *SIX1*, mutations in *SIX5* have been identified in patients with BOR that may impact DNA binding (Figure 1 and Table 1; Hoskins et al., 2007). However, more recent investigations have questioned the causative impact of *SIX5* mutations in BOR where either no mutations were detected or mutations in other genes had been identified in addition to mutations in *SIX5* (Krug et al., 2011; Wang et al., 2012; Song et al., 2013). Renal malformations are part of the complex BOR phenotype and a screen of 749 patients with congenital anomalies of the kidney and urinary tract (CAKUT) did detect one family with a mutation in *SIX5* (Hwang et al., 2014). Further research is needed to more concretely connect *SIX5* mutations as a causative factor in BOR and associated renal malformations. Another disease connected to *SIX5* expression is Myotonic dystrophy (DM1). DM1 is dominantly inherited and presents with myotonia, muscle wasting, cardiac conduction defects, fertility defects, and cataracts (Harper, 1975). The underlying genetic cause of DM1 is a CTG trinucleotide repeat expansion in the 3′ UTR of the *DMPK* gene on chromosome 19 (Brook et al., 1992; Fu et al., 1992; Mahadevan et al., 1992). The repeat expansion appears to disrupt the expression of neighboring genes, including *SIX5*, which has reduced expression in DM1 (Klesert et al., 1997; Thornton et al., 1997).

Investigation into *Six5* function in animal models has begun to tease out its role in multigenic DM1. Knock-out mice for *Six5*, both heterozygous and homozygous animals, develop cataracts with variable penetrance replicating observed DM1 phenotypes (Klesert et al., 2000; Sarkar et al., 2000). Additional studies have shown a requirement for *Six5* in spermatogonia viability and spermatozoa differentiation in male mice (Sarkar et al., 2004). Furthermore, cardiac conduction defects have been observed in heterozygous mutant mice (Wakimoto et al., 2002). One of the hallmark features of DM1 is progressive muscle wasting and hypotonia. Thorough interrogation of *Six5* function in mouse models does not support a direct role for the transcription factor in muscle phenotypes of DM1 despite expression in the developing myotome and skeletal muscle (Murakami et al., 1998; Klesert et al., 2000; Personius et al., 2005; Matynia et al., 2010). Although, triple and quadruple gene knockout of loci suspected in DM1, including *Six5*, does result in the array of multisystem defects present in myotonic dystrophy (Yin et al., 2020). Therefore, it appears the independent role of *SIX5* in DM1 is limited to the eye, spermatogonia, and cardiac conduction system.

The lack of *SIX4* mutations identified in human congenital disease is not surprising based upon studies in animal models. For example, *Six4* knock-out mice were found to be viable with no obvious developmental or progressive defects (Ozaki et al., 2001). However, *Six4* has been found to cooperate with other SIX family members in several developmental processes. Both *Six5* and *Six4* were identified to function together during vertebrate body wall development where loss of both genes resulted in omphalocele (Takahashi et al., 2018). Further mouse compound knock-out studies have uncovered *Six4* functional cooperation with *Six1* during myogenesis (Grifone et al., 2005; Relaix et al., 2013; Wurmser et al., 2020), gonadogenesis (Fujimoto et al., 2013); thymus development (Zou et al., 2006), neurogenesis (Konishi et al., 2006; Chen et al., 2009), and kidney development (Kobayashi et al., 2007; Xu and Xu, 2015). Based upon these studies, it appears *Six4* function is compensated by other family members but still plays an important supportive role during embryonic development.

The embryonic phenotypes associated with *SIX* gene manipulation in animal models or from human mutation greatly correlate with the developmental expression profile of each family member (Table 1; reviewed in Kawakami et al., 2000). However, associations with adult gene expression and disease have not been as clear. Adult expression has been detected in skeletal muscle and satellite progenitor cells for *Six1*, *Six2*, *Six4*, and *Six5* (reviewed in Maire et al., 2020). *Six1* has been detected in the adult thymus as well as the salivary gland, trachea, and at low levels in the mouse mammary gland (Ford et al., 1998; Coletta et al., 2004; Guo et al., 2011). *Six1* expression has also been demonstrated to be induced in differentiated mouse immune cells following infection (Liu et al., 2019b). *SIX2* and *SIX3* gene expression has been detected in adult pancreatic β-cells (Arda et al., 2016; Bevacqua et al., 2021) while *SIX3* and *SIX6* were found to be expressed in the adult pituitary (Aijaz et al., 2005; Xie et al., 2015). *SIX5* was detected in the epithelium of the Fallopian tube and cervix but not in the ovary or glandular epithelium (Winchester et al., 2000). Most other adult tissues appear to be negative or express very low levels of *SIX* genes including the lymph nodes, lung, and kidney (Ford et al., 1998; Kobayashi et al., 2008; Guo et al., 2011). The lack of significant expression of SIX transcription factors in adult tissue is of greater consequence in the context of cancer, where *SIX* genes become ectopically or re-expressed to drive tumorigenesis.

## Biomarkers and Prognostic Indicators in Cancer

The features of the SIX family of transcription factors that make them critical in the development of specific tissues and organ systems also makes them potentially deleterious when ectopically expressed in adult tissues. Promotion of cell proliferation or migration by SIX proteins ectopically expressed in adult tissues can and often contribute to the formation, survival, and metastasis of a variety of tumor types (Table 2).

**TABLE 2:** SIX transcription factors in cancer biology.

Gene	Associated cancer	Prognostic indication	Proposed tumorigenic function	References
SIX1	Breast	Correlation with shortened time to relapse and metastasis with lower OS	Tumor initiation, EMT, metastasis	Reichenberger et al., 2005; Micalizzi et al., 2009; Iwanaga et al., 2012
	Cervical	Potentially associated with tumor grade	Proliferation, EMT, metastasis	Liu et al., 2014b; Sun et al., 2016
	Colorectal	Correlation with lower OS	EMT, Zeb1 regulation	Ono et al., 2012
	Esophageal	Correlation with lower OS	Tumor induction, tumor cell self-renewal, TGF-β activation	Nishimura et al., 2017
	Hepatocellular carcinoma	Correlation with tumor stage, decreased OS	Increased cell proliferation, reduced apoptosis	Ng et al., 2006; Cheng et al., 2018
	Osteosarcoma	Correlation with lower OS	Cancer stem cell self-renewal	Chao et al., 2017
	Ovarian carcinoma	Correlation with lower OS	Cell proliferation, reduced apoptosis	Behbakht et al., 2007
	Pancreatic	Correlation with tumor size, stage, grade, metastasis, survival	Proliferation, migration	Li et al., 2013; Jin et al., 2014; Lerbs et al., 2017
	Papillary thyroid carcinoma	Associated with tumor stage, metastasis	Proliferation	Kong et al., 2019
	Prostate	Correlation with stage, grade, metastasis, lower OS	ND	Zeng et al., 2015
	Rhabdomyosarcoma	Correlation with lower OS	Metastasis, cell proliferation	Yu et al., 2004
	Wilms tumor	Associated with increased proliferation	Mutations change DNA binding	Wegert et al., 2015
SIX2	Breast	Correlation with lower OS	Metastasis, stem-cell self-renewal via Sox2 regulation	Oliphant et al., 2019
	Colorectal	Correlation with lower OS/tumor invasiveness	Invasiveness and drug resistance via DDX3 regulation	Wu et al., 2017
	Hepatocellular carcinoma	Correlation with lower OS	EMT via inhibition of *E*-cadherin	Li J. W. et al., 2018
	Lung	Correlation with lower OS	EMT via inhibition of *E*-cadherin	Hou et al., 2019
	Renal cell carcinoma	Correlation with lower OS	Cancer stem cell phenotype via enhanced binding to Sox2 expression	Senanayake et al., 2013; Cheng et al., 2019
	Nephroblastoma	ND	Proliferation and migration	Senanayake et al., 2013
	Wilms tumor	Unclear that Six2 is implicated in lower OS	Cancer stem cell self-renewal	Murphy et al., 2012
SIX3	Non-small cell lung carcinoma	Decreased expression correlation with lower OS	Inhibited proliferation and migration	Mo et al., 2013
	Astrocytoma	ND	Suppression of proliferation	Yu et al., 2017
	Glioblastoma	ND	Suppression of proliferation and invasion	Zhang B. et al., 2017
	Breast, prostate, stomach, esophageal, colon, lung	Decreased expression correlation with lower OS	Inhibition of EMT via lack of suppression of WNT and FOXC2	Zheng et al., 2018
SIX4	Breast	Correlation with lymph node metastasis and lower OS	Cell migration and invasion via STAT-3	Sun et al., 2020
	Colorectal	Correlation with lymph node metastasis, stage, and low OS	Proliferation	Li et al., 2017; Sun et al., 2019
	Hepatocellular Carcinoma	Correlation with microvascular invasion and metastasis with lower survival	Proliferation, EMT, metastasis	He et al., 2020
	Lung	ND	Proliferation, migration	Tang et al., 2019
SIX5	Lung squamous cell carcinoma	Correlation with lower OS	ND	Liu et al., 2016
	Ovarian tumors	ND	ND	Winchester et al., 2000
SIX6	T-cell acute lymphoblastic leukemia (T-ALL)	Correlation with poor outcomes and survival	ND	Laukkanen et al., 2020
	Non-small cell lung carcinoma	Correlation with low OS	ND	Liu et al., 2016
	Breast	Correlation with low OS	ND	Xu H. X. et al., 2016

*OS, overall survival; ND, not determined; EMT, epithelial-to-mesenchyme transition.*

The *so* subfamily (*SIX1* and *SIX2*), particularly *SIX1*, have been frequently implicated in the promotion, invasion, and survival of a variety of cancers (Blevins et al., 2015). *SIX1* alone has been shown to be overexpressed in many forms of cancer such as breast (Ford et al., 1998; Coletta et al., 2004; Iwanaga et al., 2012), ovarian (Behbakht et al., 2007), cervical (Sun et al., 2016), Wilms tumor (Wegert et al., 2015), osteosarcoma (Hua et al., 2014; Chao et al., 2017), rhabdomyosarcoma (Yu et al., 2004), and several others (Table 2). Increased levels of *SIX1* gene or protein expression was often found to be strongly correlated with poor prognosis regardless of tumor type (Blevins et al., 2015). *SIX2*, like *SIX1*, overexpression was detected in breast cancer (Wang et al., 2014; Oliphant et al., 2019) and appeared to promote increased survival, self-renewal, and metastasis of tumor cells (Table 2). All of these characteristics contribute to poor prognosis and decreased patient survival. *SIX2* has been detected in other cancers such as hepatocellular carcinoma (HCC) (Zhu et al., 2016; Li et al., 2018; Wan et al., 2019), non-small cell lung cancer (Hou et al., 2019) and colorectal cancer (Wu et al., 2017). As one might predict, increased levels of *SIX2* are highly correlated with cancers involving the kidney. *SIX2* overexpression has been identified in the pediatric cancer Wilms tumor (Murphy et al., 2012; Pierce et al., 2014; Walz et al., 2015; Wegert et al., 2015) as well as in renal cell carcinoma (Senanayake et al., 2013; Cheng et al., 2019) and nephroblastoma (Senanayake et al., 2013). Specific point mutations in *SIX1* and *SIX2* have been detected in Wilms tumor cells where they potentially increase transcriptional activity (mutations denoted in red of Figure 1; Wegert et al., 2015).

Recently, *SIX6* has been found to be associated with T-cell acute lymphoblastic leukemia (T-ALL) (Laukkanen et al., 2020), though the researchers of the study concluded that *SIX6* most likely belonged to a larger regulatory gene network and increased levels of *SIX6* alone were not sufficient to induce development of T-ALL. The study did conclude, however, that higher levels of *SIX6* was associated with inferior treatment response and poor prognosis (Laukkanen et al., 2020). Increased *SIX6* levels have also been associated with poor overall survival in non-small cell lung carcinoma and breast cancer (Liu et al., 2016; Xu H. X. et al., 2016). In stark contrast to other SIX transcription factors, increased levels of *SIX3* appear to play a tumor suppressive role rather than an oncogenic one. Higher levels of *SIX3* were associated with decreased tumor proliferation and metastasis, leading to better survival outcomes and/or prognosis in breast cancer (Zheng et al., 2018), astrocytoma (Yu et al., 2017), glioblastoma (Zhang B. et al., 2017), and lung adenocarcinoma (Mo et al., 2013).

*SIX4* expression has been detected in non-small cell lung (Tang et al., 2019), breast (Sun et al., 2020), colorectal (Li et al., 2017; Sun et al., 2019), and hepatocellular cancers (He et al., 2020). Over- or ectopic expression of *SIX4* promoted metastasis by inducing epithelial-to-mesenchymal transition and angiogenesis (Sun et al., 2019). Furthermore, significant correlations have been identified between expression levels of *SIX4*, tumor cell metastasis, and poor patient prognosis (Li et al., 2017; Tang et al., 2019; Sun et al., 2019, 2020; He et al., 2020). To date, few studies have analyzed *SIX5* function in cancer (Table 2). In one report, *SIX5* was detected in normal ovarian epithelium, as well as in malignant ovarian and borderline tumors suggesting that *SIX5* could be used as a marker for epithelial differentiation in ovarian tissue rather than a specific marker for cancer (Winchester et al., 2000). A recent meta-analysis showed that high *SIX5* expression levels correlated with poor overall survival in lung squamous cell carcinoma (Liu et al., 2016).

## Common Themes

Several common functions and modes of regulation have been identified for SIX genes, not just amongst family members, but also between roles during embryonic and cancer development (Table 3).

**TABLE 3:** Common functions and pathways of SIX family genes in development and cancer biology.

Gene	Functions in development	Functions in cancer	Common pathways	Cell cycle targets
SIX1	Progenitor cell maintenance, proliferation, cell differentiation, muscle cell migration	Proliferation, stem cell self-renewal, EMT, metastasis	Wnt/β-catenin, Notch, TGF-β	Cyclin A Cyclin D
SIX2	Progenitor cell maintenance, proliferation, cell differentiation promotion of mesenchymal phenotype	Proliferation, stem cell self-renewal, EMT, metastasis	Wnt/β-catenin, Notch, potentially TGF-β	Cyclin D
SIX3	Progenitor cell maintenance, cell differentiation	Suppression of cell proliferation; reduced EMT and tumor invasion	Wnt/β-catenin, Notch	Cyclin A
SIX4	Cooperative interaction with other SIX transcription factors	Proliferation, EMT, migration, metastasis	Potentially Wnt/β-catenin, TGF-β	Indirect regulation through c-Met
SIX5	Cell differentiation	ND	ND	ND
SIX6	Proliferation	ND	Wnt/β-catenin, Notch	P27

*See text for details and references.*

*ND, not determined.*

### Progenitor Cell Maintenance and Cell Cycle Regulation

Cell survival and proliferation are key functions of the SIX family of transcription factors. Therefore, it is not surprising that many of the gross morphological phenotypes detected from *SIX* gene mutations, either in animal studies or human syndromes, are attributed to improper maintenance of progenitor cell populations (Xu et al., 2003; Ozaki et al., 2004; Sarkar et al., 2004; Self et al., 2006; Gaston-Massuet et al., 2008; Jeong et al., 2008; Kobayashi et al., 2008; Guo et al., 2011; Wang et al., 2011; Fujimoto et al., 2013; Lu et al., 2013; Riddiford and Schlosser, 2017; Liu and Cvekl, 2017). Disruption or knock-out of *SIX* gene function in experimental studies has resulted in increased progenitor cell apoptosis concomitant with reduced proliferation in several developing tissues (Table 1). In the context of cancer, ectopic or overexpression of *SIX* genes has resulted in increased tumor cell proliferation as well as maintenance of cancer stem cells (Table 2; McCoy et al., 2009; Farabaugh et al., 2012; Cheng et al., 2019; Oliphant et al., 2019). The one exception may be *Six3* where its role in proliferation is not as clear. Overexpression of *Six3* has been shown to promote progenitor cell proliferation in the developing forebrain of zebrafish, medaka, and *Xenopus* (Kobayashi et al., 1998; Carl et al., 2002). However, other developmental studies utilizing *Six3* loss-of-function approaches have not detected expected proliferation defects (Lagutin et al., 2003; Geng et al., 2008; Liu et al., 2010; Carlin et al., 2012). It is unclear whether the observed differences in proliferation is due to experimental approach or compensation by other family members. In cancer studies, *SIX3*, appeared to function as a tumor suppressor, where overexpression in cancer cells was associated with decreased proliferation (Mo et al., 2013; Yu et al., 2017; Zheng et al., 2018). One possible explanation for the difference in cell cycle regulation between SIX3 and other family members may be attributed to different core DNA binding sequences. Six1, Six2, Six4, Six5, and Six6 have been shown to bind a TCAGGTTC core sequence identified in the *Myogenin* MEF3 promoter (Spitz et al., 1998; Harris et al., 2000; Hu et al., 2008). However, both Six3 and Six6 were found to bind to a core ATTA sequence utilized by other homeodomain containing proteins (Zhu et al., 2002; Hu et al., 2008). Six3 seems to be unique among the SIX family in transcriptional targets based upon DNA binding sequences.

The ability to regulate progenitor cell populations, both during embryonic development and in cancer, stems from the ability of SIX proteins to directly regulate the cell cycle (Table 3). Six1 has been found to transcriptionally regulate genes encoding cyclin A1 and cyclin D1 in developmental and cancer contexts (Coletta et al., 2004; Yu et al., 2006; Li et al., 2013). For the related Six2, direct interaction with cyclin promoters has not been demonstrated. However, cyclin D1 expression has been shown to be dependent upon Six2 in the developing palate (Okello et al., 2017) while studies in kidney progenitor cells have detected Six2 binding sites in the *ccnd1* (cyclin D1) promoter region (O’Brien et al., 2018). Six6, along with Dachous (Dach) proteins, promoted cell proliferation by directly repressing the expression of cyclin-dependent kinase inhibitors (Li et al., 2002; Iglesias et al., 2014) while Six4 regulated the expression of *Yap1* and *c-Met* to promote cell proliferation in HCC (He et al., 2020). Cell cycle control by SIX proteins is also accomplished via protein interactions. For example, Geminin (a cell cycle inhibitor) binding with either Six3 or Six6 inhibited cell cycle progression (Del Bene et al., 2004; Turcu et al., 2019).

Enhancing cell proliferation by SIX proteins may also occur via crosstalk with other progenitor cell markers such as Sox2. The transcriptional regulator Sox2 has been shown to be directly regulated in developmental and cancer contexts by Six1 (Zhang T. et al., 2017; De Lope et al., 2019), Six2 (Cheng et al., 2019; Oliphant et al., 2019), Six3 (Liu et al., 2006), and Six6 (Diacou et al., 2018) to further promote stem/progenitor cell phenotypes. SIX family transcription factors seem to promote progenitor cell self-renewal through both direct cell cycle regulation and via indirect mechanisms by activating additional pro-stem cell identity genes.

In parallel with regulating the cell cycle, SIX proteins also appear to influence apoptotic pathways. Loss of *SIX* function in several animal models resulted in increased progenitor cell apoptosis. Furthermore, silencing of *SIX* genes overexpressed in cancer cells resulted in increased cell death. The mechanisms of SIX regulation of apoptosis is not clear and most investigations have focused on *SIX1* in cancer cell lines. SIX1 has been shown to post-translationally regulate p53, where the levels of the two proteins are inversely related in cancer (Towers et al., 2015). Protein–protein interactions between DACH1 and SIX1, which normally behaves as a transcriptional repressor complex, can stabilize p53 levels in HCC (Cheng et al., 2018). However, DACH1 is commonly downregulated in HCC, especially in cases with high levels of *SIX1*, allowing for the reduction of p53 and cell survival. Inverse relationships have also been detected between *SIX1* and caspases. In osteosarcoma cells, overexpression of *SIX1* led to decreased caspase-3 and caspase-7 with reduced apoptosis (Yu et al., 2018). The opposite result was detected following *SIX1* knock-down where increased cell apoptosis and caspase levels were observed. Similar observations have been found in mouse trigeminal ganglia where double knock-out of *Six1* and *Six4* resulted in increased caspase-3 dependent apoptosis (Konishi et al., 2006). In addition, SIX1 and SIX4 have been shown to upregulate PI3K/AKT signaling in osteosarcoma and colorectal cancer, respectively, possibly through the downregulation of PTEN to further suppress apoptosis (Li et al., 2017; Yu et al., 2018; Na et al., 2019; Ji et al., 2020). In other studies, *SIX1* overexpression reduced TRAIL-mediated apoptosis (Behbakht et al., 2007). Taken together, *SIX* genes play critical roles in promoting progenitor cell self-renewal by directly regulating the cell cycle as well as inhibiting apoptotic pathways. However, questions remain about the mechanisms of apoptosis inhibition by SIX transcription factors such as the post-translational stabilization of p53 by a SIX1/DACH1 complex.

### Epithelial-to-Mesenchymal Transition and Cell Migration

One of the more devastating aspects of *SIX* gene overexpression in cancer appears to be from driving metastasis in part by inducing epithelial-to-mesenchymal transition (EMT). Studies in several cancer types have shown a relationship between *SIX* gene expression and increased EMT, cell migration, and tumor invasion (Table 2). A common mechanism for SIX proteins to induce EMT is through indirect regulation of *Cadherin-1* (*CDH1*), which encodes for the epithelial marker, *E*-cadherin. Increased expression of either *SIX1* or *SIX2* in several cancer types reduced the level of *CDH1* through either activating known repressors of *CDH1*, such as Zeb proteins, or by *CDH1* promoter methylation (McCoy et al., 2009; Wang et al., 2014; Li J. W. et al., 2018; Hou et al., 2019). Similar EMT-promoting mechanisms may also be present during development. Six1 appears to regulate both *N*-cadherin and *E*-cadherin in auditory epithelium (Zhang T. et al., 2017) while forced expression of genetic factors, including *Six1* and *Six2*, in kidney epithelial cells induces EMT and reduces *E*-cadherin expression (Hendry et al., 2013). Additionally, *Six2* expression was required to suppress epithelialization of renal progenitor cells and *Six2* null embryonic mouse kidney explants showed expanded *E*-cadherin expression, suggesting regulation by the transcription factor (Self et al., 2006; McMahon, 2016). Conversely, continual expression of *Six2* in renal progenitor cells *in vivo* inhibited *Cdh1* expression and mesenchymal-to-epithelial transition (Chung et al., 2016). *SIX* genes also appear to activate pathways known to promote EMT and cell migration such as the c-Met/HGF pathway. One of the downstream targets of c-Met/HGF is Snail, a known repressor of *E*-cadherin (Wang et al., 2020). It has been demonstrated that SIX4 could directly activate c-Met expression in HCC providing a mechanism for promoting EMT, cell migration, and metastasis (He et al., 2020). In support of the finding in HCC, both Six1 and Six4 have been shown to activate *met* expression in both embryonic mouse and zebrafish and this activation was required for skeletal muscle precursor cell migration (Grifone et al., 2005; Talbot et al., 2019).

### Signal Transduction Pathways

SIX protein function has been connected to a number of regulatory and signal transduction pathways, however, interactions with Wnt, Notch, and TGF-β pathways appear to be shared most amongst family members in both development and cancer (Table 3). Wnt signaling regulation has been connected to *Six1*, *Six2*, *Six3*, *Six4*, and *Six6*. *Six1* overexpression upregulated Wnt pathway genes and promoted β-catenin nuclear localization in mammary gland tumors and colorectal cancer cell lines (McCoy et al., 2009; Song et al., 2019). In developing auditory sensory epithelium, Six1 binding sites indicative of gene activation have been detected upstream of *Wnt5a* and other Wnt targets (Li et al., 2020). During kidney development, Wnt/beta-catenin and Six2 have opposing functions of cell differentiation and self-renewal, respectively (Park et al., 2007). Wnt/beta-catenin repressed *Six2* expression in renal progenitor cells to help control mesenchymal-to-epithelial transition (Park et al., 2012). A similar opposing interaction has been observed in the pediatric kidney tumor, Wilms tumor. *Six2* overexpression in Wilms tumor cells resulted in downregulation of Wnt pathway genes (Pierce et al., 2014). However, Six2 has been shown to be activated by Wnt in limb tendon precursor cells suggesting context dependent regulation (Yamamoto-Shiraishi and Kuroiwa, 2013). In the developing forebrain, Six3 directly repressed the expression of *Wnt1* as well as *Wnt8b* (Lagutin et al., 2003; Liu et al., 2010). Repression of *Wnt1* by Six3 has been detected in mammary glands as well as breast cancer cells (Kumar et al., 2010). Both Six3 and Six6 suppressed Wnt signaling during retinal development to maintain retinal progenitor cells (Diacou et al., 2018). In breast cancer, *Six3* was transcriptionally targeted for repression by metastatic tumor antigen 1 (MTA1) which in turn upregulated Wnt1 (Kumar et al., 2010). Wnt signaling has been shown to play a significant role in cancer stem cell maintenance and metastasis (Zhan et al., 2017) and repression of Wnt by Six3 supports its role as a tumor suppressor. Finally, repression of *Six4* by Wnt signaling has been implicated in neuronal placode development in the chick model (Litsiou et al., 2005).

Notch signaling and SIX transcription factor function have been associated in different developmental and cancer contexts. Six1, for example, has been shown to regulate Notch pathway targets *hes8* and *neurog1* during *Xenopus* neurogenesis (Riddiford and Schlosser, 2017) as well as *jagged1* in mouse mandibular arch formation (Tavares et al., 2017). In breast cancer cells, Notch signaling was found to be upregulated with *Six1* overexpression (Smith et al., 2012). Six1 also appeared to be a downstream effector of Notch2 in the developing olfactory epithelium and in lung adenocarcinoma cells (Rodriguez et al., 2008; Mimae et al., 2012). Overexpression of both *SIX1* and *NOTCH2* in lung cancer was associated with poor overall survival (Mimae et al., 2012). Further supporting a SIX/Notch pathway, *Six2* has been shown to be regulated by Notch in kidney progenitor cells during renal organogenesis (Chung et al., 2016). Additionally, Notch1 expression is dependent upon both Six3 and Six6 in retinal development (Diacou et al., 2018) and expression of human *SIX6* glaucoma risk alleles in *Xenopus* embryos downregulated the Notch pathway (Teotia et al., 2017).

Compelling evidence has emerged connecting the TGF-β pathway with *SIX* gene overexpression in various cancers. For example, *SIX1* overexpression in breast cancer cell lines activated TGF-β signaling and activity of both factors correlated with poor prognosis in breast cancer (Micalizzi et al., 2009). Further investigation showed that SIX1 could bind to the promoter of *TBRI* and regulate its transcription (Micalizzi et al., 2010). The Six1/TGF-β pathway appears to switch cells toward a pro-EMT fate, an important step toward tumor metastasis (Micalizzi et al., 2009; Farabaugh et al., 2012; Smith et al., 2012). Similar interactions have been detected in models of cervical cancer and esophageal squamous cell carcinoma (Liu et al., 2014a; Nishimura et al., 2017). To date, SIX/TGF-β networks have not been thoroughly studied during embryonic development. However, components of the TGF-β pathway were found to be downregulated in *Six1*/*Six4* double knockout mouse Pax7 + muscle precursor cells (Wurmser et al., 2020) while *Six2* expression in metanephric mesenchyme progenitor cells may be controlled by TBRII/Smad3 (Mao et al., 2017).

### Activation/Repression Functions

SIX family transcriptions factors can behave as transcriptional activators or repressors. For example, mouse Six1 has been shown to activate gene expression in skeletal muscle cells (Li et al., 2003) and human SIX1 functioned in HCC as either an activator or repressor of gene expression (Cheng et al., 2018). The use of ChIP-seq has demonstrated that Six2 in mouse kidney progenitor cells functioned as a gene activator to promote progenitor cell self-renewal or as a repressor to inhibit cell differentiation (O’Brien et al., 2018). Six3 was found to repress *AURKA* and *AURKB* genes in astrocytoma cells (Yu et al., 2017) while the transcription factor was found to activate the expression of *rhodopsin* in the mouse retina (Manavathi et al., 2007). What determines the activation or repression function of the SIX transcription factors appears to the presence of interacting proteins such as Eya, Dach, and Grg. Eya proteins have been shown to bind to Six1, Six2, Six4, Six5, and Six6 to promote gene activation (Ohto et al., 1999; Ikeda et al., 2002; Li et al., 2003; Hu et al., 2008; Xu J. et al., 2016). Interestingly, Six3 has not been demonstrated to interact with Eya proteins (Zhu et al., 2002). In contrast to activation SIX/Eya complexes, interactions with Dach function to repress gene transcription. For example, Six6/Dach interactions have been demonstrated to act as a repressor complex in mouse retina and pituitary gland (Li et al., 2002). Although, the presence of Eya proteins can convert the repressive function of SIX/Dach complex toward gene activation (Li et al., 2003). Similarly, interactions demonstrated between Six3 or Six6 with Grg acted as repressor complexes (Kobayashi et al., 2001; Lopez-Rios et al., 2003). Zebrafish Six2 and Six4 were also found to bind to Grg proteins suggesting conservation of the repressor complex (Kobayashi et al., 2001). In cell culture experiments, Six3 was shown to activate promoter sites when binding alone, however, in the presence of Geminin, the two proteins complexed and repressed promoters (Del Bene et al., 2004). *Eya*, *Grg*, and *Dach* are commonly co-expressed with *SIX* transcription factors during development. The determination of whether the transcriptional complexes promote or repress gene expression remains unclear. Of clinical significance, repressive factors such as *DACH* are commonly downregulated in cancer while *EYA* genes are overexpressed (reviewed in Blevins et al., 2015; Kingsbury et al., 2019) which may provide a permissive environment for SIX dependent tumor growth and metastasis.

### Transcriptional and Epigenetic Regulation

Appropriate control of *SIX* gene expression is required for normal tissue development and homeostasis. However, how members of the SIX family are regulated in development and reactivated in cancer remains unclear. Several studies have interrogated the upstream promoters of *SIX* genes to gain insight into their regulation. Binding sites for several transcription factors have been identified in the *Six1* promoter which included, Sox, Pax, Fox, Tcf/Lef, Smad, E-box binding basic helix-loop-helix, and nuclear hormone receptor proteins (Sato et al., 2012; Sato et al., 2015). Additional promoter characterization for *Six1* detected consensus sites for MyoD, Creb, and Pax7 (Wei et al., 2017). *Six2* was shown to be regulated by Hox proteins, Hoxa2 (Kutejova et al., 2008; Yallowitz et al., 2009) and Hox11 (Yallowitz et al., 2009; Park et al., 2012; O’Brien et al., 2018) as well as β-catenin, Wilms tumor 1 and Odd-skipped related 1 (Park et al., 2012; O’Brien et al., 2018). Both Six1 and Six2 are capable of autoregulation and Six2 binding was detected on the *Six1* promoter (Brodbeck et al., 2004; O’Brien et al., 2016). The presence of Tcf/Lef, β-catenin, and smad binding sites in the *Six1* and *Six2* promoters supports experimental evidence of Wnt and TGF-β regulation of SIX function (Table 3). Pax6 has been demonstrated to activate both *Six3* and *Six6* expression (Goudreau et al., 2002) along with other activators such as Prox1, Sox2, Sox3, and Lhx2 (Lengler and Graw, 2001; Tetreault et al., 2009; Lee et al., 2012). Six3 has been shown to autorepress its expression along with other repressor proteins including Msx2 and MTA1 (Lengler and Graw, 2001; Manavathi et al., 2007). *Six6* promoter repression has been demonstrated for FoxD1 and Onecut (Ledford et al., 2017). Despite these findings, a great deal remains to be learned about the regulation of *SIX* genes during organ development and especially how these genes become reactivated in cancer cells.

Experimental evidence has suggested a significant role for epigenetic regulation in controlling *SIX* gene expression. Two emerging epigenetic mechanisms appear to be DNA methylation and targeting with microRNA (miRNA). Differential methylation patterns have been identified during embryonic development and in cancerous tissues for *Six2*, *Six3*, *Six5*, and *Six6* (Table 4). For pro-oncogenic functions of *Six2* and *Six6*, hypomethylation of promoter and genic regions correlated with increased expression (Marcinkiewicz and Gudas, 2014; Song et al., 2015; Berdasco et al., 2017; Sun et al., 2018). Two reports have associated hypermethylation of *SIX6* with cancer types, however, expression levels of *SIX6* were not addressed and it remains unclear the significance of gene methylation in these instances (Zhao et al., 2013; Lindqvist et al., 2014). Reduced *SIX5* expression was associated with increased DNA and repressive-associated histone methylation linked to trinucleotide expansion in DM1, suggesting a similar epigenetic mechanism to control *SIX* gene expression (Filippova et al., 2001; Yanovsky-Dagan et al., 2015). For *SIX3*, the tumor suppressor appeared to be hypermethylated in lung cancer and glioblastoma accounting for decreased expression in these cancer types (Mo et al., 2013; Yu et al., 2020).

**TABLE 4:** Epigenetic regulation of SIX transcription factor genes.

Gene	Epigenetic mechanism	Identified system	References
Six1	miR-448-5p	TGF-β induced lung fibrosis in asthma	Yang et al., 2019
	mirR-23a	Endometrial cancer	Li et al., 2019
	miR-30a	Prostate cancer	Zhu et al., 2016
	miR-488	Ovarian cancer	Yang et al., 2017
	miR-548a-3p	Warburg effect; breast cancer	Li L. et al., 2018
	miR-362	Cervical cancer	Shi and Zhang, 2017
	miR-185	Ovarian cancer, pediatric renal tumors, breast cancer	Imam et al., 2010
	miR-30a	Zebrafish skeletal muscle development	O’Brien et al., 2014
Six2	miR-335-5p	Breast cancer	Jia et al., 2020
	miR-181b	Kidney progenitors	Lyu et al., 2013
	Hypomethylation	Wilms tumor	Song et al., 2015
	Hypomethylation	Hepatic cell carcinoma	Sun et al., 2018
	Hypomethylation	Oral squamous cell carcinoma	Marcinkiewicz and Gudas, 2014
	Histone methylation associated with gene activation	Adult kidney epithelial cells	Omer et al., 2013
Six3	miR-196a	*Xenopus* eye development	Qiu et al., 2009
	Hypermethylation	Glioblastoma, astrocytoma	Yu et al., 2017, 2020
	Hypermethylation	Lung cancer	Mo et al., 2013
Six4	miR-384	Gastric cancer	Liu et al., 2020
	miR-203a	Bladder cancer	Na et al., 2019
	miR-621	Non-small cell lung carcinoma	Zhang et al., 2019
Six5	Increased repressive histone methylation and DNA hypermethylation	DM1	Filippova et al., 2001; Yanovsky-Dagan et al., 2015
Six6	Hypermethylation	Non-small cell lung carcinoma	Zhao et al., 2013
	Hypermethylation	Breast cancer	Lindqvist et al., 2014
	Hypomethylation	Retinal progenitor cells	Berdasco et al., 2017

In addition to DNA methylation, several miRNAs have been identified as epigenetic regulators that down regulate *SIX* expression (Table 4). Much of the research focus has been on various cancer cell lines and tumor types where miRNAs that normally target and suppress *SIX* mRNA transcripts were downregulated, allowing for SIX transcription factor induced cell proliferation and EMT. Several miRNAs have been identified that target *Six1*, *Six2*, and *Six4* in a diverse array of cancers (Table 4). In addition to the cancer studies, examples of miRNA regulated *SIX* gene expression have been detected during embryonic development. *Six1* was found to be directly regulated by miR30a during zebrafish skeletal muscle development (O’Brien et al., 2014). The related *Six2* has been shown to be regulated by miR-181b in cultured metanephric mesenchyme cells and the interaction may function to control cell differentiation (Lyu et al., 2013). Finally, *Six3* may potentially be regulated by miRNAs during eye development, although these results need to be further investigated to confirm this mechanism (Qiu et al., 2009).

## Future Directions

The fields of developmental biology and cancer research together have provided great insight into the important functions of the *SIX* gene family in vertebrates. However, many questions remain including how the gene family is regulated, the regulation of activation or repression complexes, and whether SIX proteins are viable therapeutic targets. Efforts have been made to identify transcriptional regulators and characterize the promoters of *SIX* genes. For example, complex ChIP-seq analysis identified regulatory regions of *Six2* in kidney progenitor cells and how Six2 may function in complex transcriptional regulatory networks (Park et al., 2012; O’Brien et al., 2018). The functional importance of the putative *Six2* regulatory sequences and how they function in the balance between kidney progenitor cell maintenance and differentiation remains to be determined. It is also unclear whether any of the identified transcription factor binding sites upstream of *Six2* or other *SIX* family members become re-engaged in cancer. In addition to the specific proteins that regulate *SIX* family enhancers and promoters, it is of interest to further identify upstream signal transduction pathways that can induce *SIX* gene expression. Evidence has shown a role for Notch, Wnt, and TGF-β pathways to not only be targets of SIX proteins but also to feedback onto *SIX* genes (Table 3). Identification of upstream pathways may be helpful in the context of congenital disease where clinical manifestations are commonly the result of *SIX* gene haploinsufficiency and compensation by wild-type alleles could reduce disease severity. Upstream activation pathways of *SIX* expression would also be of interest in cancer where they could provide new opportunities to reduce SIX induced tumorogenesis. Another tumorogenic target to reduce SIX function would be miRNAs, which have been found to be reduced in several SIX family associated cancers (Table 4). MicroRNAs appear to be an intriguing class of therapeutic targets and reintroduction into cancer cells may allow for specific downregulation of SIX expression in cancer (Rupaimoole and Slack, 2017).

A second area to further investigate is the regulation of activator vs. repressor complexes involving SIX proteins and other conserved factors such as Eya and Dach proteins. During development, these three protein families are commonly co-expressed to maintain a balance between progenitor cell proliferation and differentiation. Does the presence of Eya proteins always promote transcriptional activation even in the presence of repressors such as Dach (Li et al., 2003) or is there more complex regulation of additional SIX binding factors involved. Efforts have begun to better elucidate how SIX factors coordinate with other transcription factors to regulate target genes (O’Brien et al., 2018; Ogawa et al., 2019; Xu et al., 2021). The mechanisms regulating SIX transcriptional activation or repression would provide great insights for both developmental and cancer biology.

Traditionally, transcription factors have been seen as poor drug targets despite the central role they can play in disease such as cancer (Bushweller, 2019). Past difficulties have included targeting protein-DNA or protein-protein interactions due to the charge and flat shape of binding surfaces (Arkin et al., 2014). However, great progress has been made in better understanding protein structure, identifying the residues required for protein interactions, and the regulation of protein function through post-translational modifications. All of these areas are potential targets to control SIX transcription factors in the context of cancer (Bushweller, 2019). One approach has already been demonstrated in a breast cancer model where a small molecule inhibited the interaction between SIX1 and EYA2, reducing downstream TGF-β signaling and EMT leading to reduced metastasis in mouse xenografts (Zhou et al., 2020). Further exploration should identify additional novel regulators of SIX protein function and in conjunction with other therapeutic modalities, such as epigenetic modifiers, could prove effective strategies to combat SIX induced tumorogensis. Such therapeutic insights will only arise from the continual integration of developmental and cancer biology research into the function of the SIX family of transcription factors.

## Author Contributions

LM, LF, and TC conceived and developed the manuscript outline, and wrote and edited the manuscript. LM, LF, BB, and TC researched the literature and developed the tables. LM and TC created the figure. All the authors contributed to the article and approved the submitted version.

## Conflict of Interest

The authors declare that the research was conducted in the absence of any commercial or financial relationships that could be construed as a potential conflict of interest.

## Publisher’s Note

All claims expressed in this article are solely those of the authors and do not necessarily represent those of their affiliated organizations, or those of the publisher, the editors and the reviewers. Any product that may be evaluated in this article, or claim that may be made by its manufacturer, is not guaranteed or endorsed by the publisher.
